# Characterization of the Spatiotemporal Localization of a Pan-Mucorales–Specific Antigen During Germination and Immunohistochemistry

**DOI:** 10.1093/infdis/jiae375

**Published:** 2024-08-10

**Authors:** Alyssa C Hudson, Dora E Corzo-Léon, Iana Kalinina, Duncan Wilson, Christopher R Thornton, Adilia Warris, Elizabeth R Ballou

**Affiliations:** Medical Research Council Centre for Medical Mycology, University of Exeter; Department of Microbiology, Royal Devon University Hospitals NHS Foundation Trust; Medical Research Council Centre for Medical Mycology, University of Exeter; Medical Research Council Centre for Medical Mycology, University of Exeter; Medical Research Council Centre for Medical Mycology, University of Exeter; Biosciences, Faculty of Health and Life Sciences, University of Exeter; ISCA Diagnostics Ltd, Hatherly Laboratories, Exeter, United Kingdom; Medical Research Council Centre for Medical Mycology, University of Exeter; Medical Research Council Centre for Medical Mycology, University of Exeter

**Keywords:** fungal cell wall, invasive fungal infection, monoclonal antibody, Mucorales, mucormycosis

## Abstract

**Background:**

Mucormycosis is an aggressive invasive fungal infection caused by molds in the order Mucorales. Early diagnosis is key to improving patient prognosis, yet it relies on insensitive culture or nonspecific histopathology. A pan-Mucorales–specific monoclonal antibody (mAb), TG11, was recently developed. Here, we investigate the spatiotemporal localization of the antigen and specificity of the mAb for immunohistochemistry.

**Methods:**

We used immunofluorescence microscopy to assess antigen localization in 11 Mucorales species of clinical importance and live imaging of *Rhizopus arrhizus* germination. Immunogold transmission electron microscopy revealed the subcellular location of mAb TG11 binding. Finally, we performed immunohistochemistry of *R arrhizus* in an ex vivo murine lung infection model alongside lung infection by *Aspergillus fumigatus*.

**Results:**

Immunofluorescence revealed TG11 antigen production at the emerging hyphal tip and along the length of growing hyphae in all Mucorales except *Saksenaea*. Time-lapse imaging revealed early antigen exposure during spore germination and along the growing hypha. Immunogold transmission electron microscopy confirmed mAb TG11 binding to the hyphal cell wall only. The TG11 mAb stained Mucorales but not *Aspergillus* hyphae in infected murine lung tissue.

**Conclusions:**

TG11 detects early hyphal growth and has valuable potential for diagnosing mucormycosis by enhancing discriminatory detection of Mucorales in tissue.

## Teaser

A pan-Mucorales antibody binds to germinating hyphae and can discriminate Mucorales and *Aspergillus* hyphae by immunohistochemistry.

Mucormycosis is an aggressive invasive fungal infection caused by molds in the order Mucorales. These are ubiquitous environmental fungi, found globally in soil and on decaying organic matter. Over 20 fungal species within the Mucorales group are known to cause infection in humans, the most common of which is *Rhizopus arrhizus* [1–7]. While considered a rare infection, mucormycosis is the second-most common invasive mold infection in children and adults after aspergillosis [8, 9]. The main risk factors are diabetes mellitus, hematologic malignancy, and solid organ transplant, yet a range of conditions predisposes to mucormycosis, including corticosteroid use, neutropenia, renal failure, and deferoxamine therapy [1, 3, 6, 7, 10–12]. Traumatic wounds and burns are common risk factors in individuals who are otherwise immunocompetent [6, 7, 10, 12]. Incidence is rising globally, likely due to the growing prevalence of diabetes mellitus, increased use of immunosuppressive therapies, and use of azole antifungal prophylaxis in patients who are immunosuppressed [5, 7, 13–15]. COVID-19 is the most recently identified risk factor and was associated with an unprecedented surge in cases of mucormycosis worldwide, particularly in India, where >47 500 cases were reported during a 2-month period in 2021 [2, 4, 16, 17]. Clinical presentation depends on the site of infection, which is most commonly sino-orbital, pulmonary, or cutaneous [1, 3, 5, 7, 10]. A consistent feature is aggressive disease with rapid and destructive growth, extensive angioinvasion, and subsequent tissue necrosis and infarction. Contiguous spread is common, and hematogenous dissemination occurs in up to 23% of cases [7, 10, 12]. Overall mortality is high, at approximately 50%, and reaches >90% in disseminated infection [1, 3, 6, 7, 10, 12].

Due to the rising incidence of mucormycosis, consistently high mortality, and urgent need for improved diagnostics, Mucorales are ranked as high-priority pathogens by the World Health Organization [18]. Timely diagnosis of invasive infection, allowing prompt and appropriate treatment, is key to improving patient prognosis [19]. As compared with other mycoses, the development of non–culture-based diagnostic tools to identify mucormycosis has been neglected. Screening and early diagnosis of invasive fungal infection in high-risk groups via antigen-based tests that can be performed on blood or other clinical specimens, such as pan-fungal 1-3-β-D-glucan, *Aspergillus* galactomannan, and cryptococcal antigen, offer important adjuncts to histopathology and culture. However, besides ruling out other invasive fungal infections, these are not appropriate for diagnosis of mucormycosis due to the low presence of these antigens in the Mucorales cell wall [17]. Instead, diagnosis continues to rely on histopathology by using nonspecific stains to visualize fungal elements in tissue samples and by insensitive culture of fungi from tissue biopsies or bronchoalveolar lavage fluid to confirm identification [17, 20, 21].

Thornton et al recently developed a murine IgG2b monoclonal antibody (mAb), TG11, that is pan-Mucorales specific [22]. TG11 was raised against extracellular polysaccharide from *Lichtheimia corymbifera* and binds to extracellular polysaccharide antigen secreted by all Mucorales fungi but not unrelated molds and yeasts, including *Aspergillus*, *Candida*, *Cryptococcus*, *Fusarium*, *Lomentospora*, and *Scedosporium* species [22]. TG11 has been incorporated into a lateral flow device to allow the rapid detection of Mucorales from serum or bronchoalveolar lavage fluid [22], yet the spatiotemporal expression and in vivo relevance of this antigen remain undescribed. Here, we investigate the potential of this mAb to differentiate infection from colonization or sample contamination with environmental spores and to specifically detect invasive disease in murine tissue samples. Specifically, we investigate the dynamics of the antigen recognized by TG11 during spore germination and invasive growth in tissue, as well as the ability of TG11 mAb to differentiate invasive mucormycosis from invasive aspergillosis, the most relevant clinical differential diagnosis. We characterize the spatiotemporal localization of the TG11 antigen in the most important causative species of mucormycosis with immunofluorescence and immunogold microscopy. Furthermore, we use an ex vivo murine lung infection model to demonstrate the utility of TG11 in visualizing invasive hyphal growth in histology sections. Taken together, our work shows that TG11 detects early spore germination and hyphal growth in vitro and in an ex vivo murine lung infection model, and it presents a novel opportunity to detect invasive infections by Mucorales fungi in tissue samples, differentiated from invasive *Aspergillus* infections.

## MATERIALS AND METHODS

### Ethics Statement (Mouse Tissue)

All animal experiments were conducted in compliance with the United Kingdom Home Office licenses for research on animals and approved by the University of Exeter Ethical Review Committee. Ex vivo lung infections were performed with uninfected CD1 female mice scheduled to be culled, in keeping with 3Rs objectives to reduce the number of animals involved in research. All experiments were performed at the University of Exeter (establishment license X7C5DF140).

### Spore Harvesting

Fungal spores (Table 1) were cryopreserved at −80 °C in YPG broth (0.3% yeast extract, 1% peptone, 2% glucose) containing 50% glycerol and cultured on YPG agar (YPG broth + 2% agar) 30 °C, for 5 to 7 days to induce sporulation. *Apophysomyces variabilis* was cultured on Czapek Dox agar (CM0097; Oxoid), 30°C, for 14 days. Spores were harvested from mycelia in 10 mL of sterile 1× phosphate-buffered saline (PBS; BR0014G, Oxoid Phosphate Buffered Saline Tablets–Dulbecco A) with an L-shaped spreader; after which, spore suspensions were filtered through a 40-μm sterile strainer to exclude hyphae and centrifuged (4000 rpm, 5 minutes) with supernatants discarded. The pellet was resuspended in 5 mL of PBS and spores quantified with a Neubauer hemocytometer. Spores were routinely stored at 4 °C for up 14 days.

**Table 1.** Mucoralean Fungal Strains Used in This Study.

Species	Isolate No.	Origin	Source
*Rhizopus arrhizus* var *delemar*	RA 99-880	Reference strain^a^	ATCC
*Rhizopus arrhizus* var *arrhizus*	MRL 21E307	Clinical isolate	UKHSA
*Rhizopus microsporus*	FP469-12	Clinical isolate^b^	QEHB^c^
*Mucor circinelloides*	MRL 22E56177	Clinical isolate	UKHSA
*Lichtheimia corymbifera*	MRL 20E42327	Clinical isolate	UKHSA
*Lichtheimia ramosa*	MRL 21E1059	Clinical isolate	UKHSA
*Cunninghamella bertholletiae*	CBS 151.80	Clinical isolate	CBS
*Apophysomyces variabilis*	CBS 658.93	Clinical isolate	CBS
*Rhizomucor pusillus*	MRL 22E19013	Clinical isolate	UKHSA
*Saksenaea erythrospora*	CBS 138279	Clinical isolate	CBS
*Syncephalastrum contaminatum*	MRL 22E22635	Clinical isolate	UKHSA
*Aspergillus fumigatus*	A1160^+^	Clinical isolate^d^	FGSC

Species are listed in order of the frequency with which they are isolated from clinical cases of mucormycosis [1–7].

Abbreviations: ATCC, American Type Culture Collection; CBS, Westerdijk Fungal Biodiversity Institute; FGSC, Fungal Genetics Stock Center; QEHB, Queen Elizabeth Hospital, Birmingham; UKHSA, UK Health Security Agency (National Collection of Pathogenic Fungi).

^a^Strain RA 99-880 was originally a clinical isolate from Ma et al [23] and is a widely used reference strain.

^b^Strain FP469-12 is a clinical isolate from Itabangi et al [24].

^c^Isolated by Deborah Mortiboy.

^d^Strain A1160p^+^ was derived from a clinical isolate from Fraczek et al [25, 26].

### Cell Preparation, Fixation, and Staining for Indirect Immunofluorescence Microscopy

The pan-Mucorales–specific mAb TG11 and the *Aspergillus*-specific mAb JF5 [27] were gifts from ISCA Diagnostics Limited. Spores were inoculated at 10^7^ spores/mL in 5-mL HL5 medium with glucose broth (HLG0101; Formedium Ltd) and incubated at 37 °C (150 rpm) for up to 8 hours. Swollen spores and germlings were pelleted (4000 rpm for 5 minutes). For hyphal cultures, cells were collected and pelleted at 13 000 rpm for 5 minutes. Pellets were washed in 1× PBS (13 000 rpm, 5 minutes), resuspended in 4% methanol-free formaldehyde in PBS, and incubated on a roller for 1 hour at room temperature (RT). Following incubation, cells were pelleted (13 000 rpm, 5 minutes), formaldehyde removed, and cells washed twice with 1 mL of PBS as indicated earlier. Pellets were resuspended in 1 mL of 5% bovine serum albumin (BSA) in 0.1% Tween 20 1× PBS (PBST) and incubated on a roller for 30 minutes at RT. Cells were pelleted at 13 000 rpm for 5 minutes and supernatant removed; after which, they were resuspended and incubated in 0.5 mL of 5-µg/mL TG11 in 1% BSA in 0.1% PBST for 1 hour at RT on a roller. The cells were pelleted (4000 rpm, 5 minutes), antibody solution removed, and washed 3 times with 1 mL of PBS. Pellets were incubated for 1 hour at RT in 1:1000 Cy5-conjugated goat anti-mouse polyclonal IgG (ab6563; Abcam) in 1% BSA in 0.1% PBST with 10 µg/mL of calcofluor white (CFW); after which, they were pelleted (4000 rpm, 5 minutes), washed 3 times with 1 mL of PBS, and resuspended in 1× PBS. For the germination timeline, *R arrhizus* var *delemar* spores were inoculated into 17.5 mL of HL5 broth (37 °C, 150 rpm), sampled in 2-mL aliquots hourly, and processed as indicated earlier. Immunoassay controls were performed with *R arrhizus* var *delemar* cultured for 4 hours as described previously: positive control with CFW (A+), positive control without CFW (A–), primary only (B), isotype control (C), secondary only with CFW (D+), secondary only without CFW (D–), unstained (E). The isotype control BD6, a murine IgG2b against human Dectin-1 (from Dr Janet Willment, University of Exeter), was prepared at 5 µg/mL in 1% BSA in 0.1% PBST. Images were acquired by an inverted DeltaVision Elite microscope (60× with DAPI, FITC, and Cy5 channels; IMSOL) and acquired as Z-stacks every 0.2 μm for at least 10 μm.

### Conjugation of Cy5 to TG11 and BD6

Cy5 was conjugated to TG11 and the BD6 isotype control antibody with a Lightning-Link Rapid Cy5 Antibody Labeling Kit (AB188288-1003; Bio-Techne Ltd). Conjugation was performed per the manufacturer's instructions with 0.5 mg/mL of antibody.

### Direct Immunofluorescence Time-lapse Microscopy of Live Cells in Microfluidic Chambers

Spores were inoculated (10^7^ spores/mL) in YPD and incubated (37 °C, 150 rpm) for 2.5 hours to swell; then, they were diluted 1:10 in YPG and loaded into a custom-fabricated polydimethylsiloxane microfluidic chamber with U-shaped traps with a height of 10 µm for imaging on a prewarmed (37 °C) inverted DeltaVision Elite microscope. The chip was perfused at 1 µL/min with 1XYNB without amino acids and 3% glucose, 1% BSA, 1-µg/mL CFW, and 1-µg/mL TG11-Cy5 and the cells imaged every 20 minutes for 3 hours.

### Immunogold Transmission Electron Microscopy

#### Conjugation of Gold Particles to the Secondary Antibody

Goat anti-mouse polyclonal IgG (0.1 mg/mL, ab7063; Abcam) was directly conjugated to 40-nm gold particles with a gold conjugation kit (ab154873; Abcam) per the manufacturer's instructions.

#### Preparation of Cells for Transmission Electron Microscopy

*R arrhizus* var *delemar* 99-880 spores were inoculated in 5 mL of YPG broth at 10^7^ spores/mL, incubated at 37 °C (150 rpm) for 4 hours, and pelleted as indicated earlier. Samples were fixed in 4% formaldehyde in cacodylate buffer and then dehydrated in 30%, 50%, 70%, 95%, and 100% graded ethanol. After LR White infiltration (London Resin; Agar Scientific), samples were polymerized at 60 °C in an oxygen-free environment, and 60-nm sections were mounted on copper grids.

#### Immunostaining and Contrast Staining for Transmission Electron Microscopy

Grids were incubated in a series of 100-µL reagent droplets beaded on parafilm and transferred by a perfect loop. Grids were washed serially with 70% ethanol and sterile deionized water between transfers. Grids were blocked with 1% BSA, 0.5% Tween 80, in PBS for 20 minutes; washed 3 times for 5 minutes in 0.1% BSA in PBS (incubation buffer); and stained in 5-µg/mL mAb TG11 in incubation buffer for 90 minutes (negative control, incubation buffer alone; IgG2b isotype control, 5-µg/mL BD6 in incubation buffer). Grids were washed 6 times for 5 minutes in incubation buffer and stained with gold-conjugated goat anti-mouse IgG (50 µL, 1 µg/mL) in incubation buffer for 60 minutes. Grids were washed 6 times in incubation buffer, 3 times in PBS, and 3 times in sterile deionized water (5 minutes each). Grids were air-dried on filter paper and then contrast stained with a 2% aqueous solution of uranyl acetate for imaging.

#### Sampling of Immunogold-Labeled Cells and Gold Density Calculations

Ten images containing cells from a single section from the TG11 and IgG2b grids were acquired by a systematic random sampling method from the bottom right-hand corner of each grid, moving systematically across sections at regular intervals independent of image content at ×3000 magnification. Spore and hyphal cell walls were distinguished visually by the presence of a thickened outer layer. Gold particles detected in the total background area, cell area, and cell walls of spores and hyphae were counted as a proxy for TG11 binding. Gold density was calculated by dividing the total area or length of the cell or field (background) by the number of gold particles.

### Direct Immunofluorescence Microscopy of Murine Lung Tissue Infected With *R arrhizus* and *Aspergillus fumigatus*

Female CD1 mice culled by CO_2_ inhalation were immediately infected intratracheally with 50 µL of PBS containing 10^4^  *R delemar* 99-880 spores, 10^4^  *A fumigatus* A1160p^+^ spores, or PBS alone. Lungs were harvested and washed in sterile PBS and then incubated for 24 hours at 37 °C and 5% CO_2_ in DMEM/F12 medium (A4192001; Gibco), 1% fetal bovine serum (A5256801; Gibco), and 1% penicillin/streptomycin (15140-122; Gibco). Recovered lungs were embedded in OCT medium (Lamb/OCT; Thermo Scientific), fast frozen with dry ice and isopentane, and stored at −80 °C until sectioned via a cryostat (CM1521; Leica Biosystems). The 6-µm slices mounted on super-frost glass slides (J1800AMNZ; Epredia Inc) were fixed with 4% methanol-free formaldehyde for 1 hour and stored at −20 °C in 70% ethanol in a Coplin jar. For staining, samples were permeabilized with 0.2% Triton X-100 in PBS for 20 minutes, washed 3 times with PBS, and blocked with 5% BSA in 0.1% PBST for 30 minutes; after which, they were stained with 5-µg/mL TG11-Cy5 conjugate or 5-µg/mL JF5-Cy5 conjugate plus 1× SYBR Green nucleic acid stain (S7563; Invitrogen) in 1% BSA in 0.1% PBST and incubated overnight at 4 °C while covered in the dark before counterstaining with 10-µg/mL CFW. Slides were washed with PBS and dried in the dark at RT; then, coverslips were mounted with 5 µL of 90% glycerol in PBS. Infected controls were 1× SYBR Green nucleic acid stain and 10-µg/mL CFW in 1% BSA in 0.1% PBST. Isotype control was 5-µg/mL Dectin-1/CLEC7A BD6-Cy5 mAb (BioRad MCA4662GA). Images were acquired on a DeltaVison Elite microscope. For ex vivo sections, images were acquired every 0.2 μm for 16.8 μm.

### Image Analysis

All images were analyzed with Fiji ImageJ [28]. Germination micrographs and tissue sections are presented as summed Z-projections.

## RESULTS

### TG11 Antigen Is Localized Primarily to Growing Hyphae in *R arrhizus* var *delemar*

**Figure 1.**
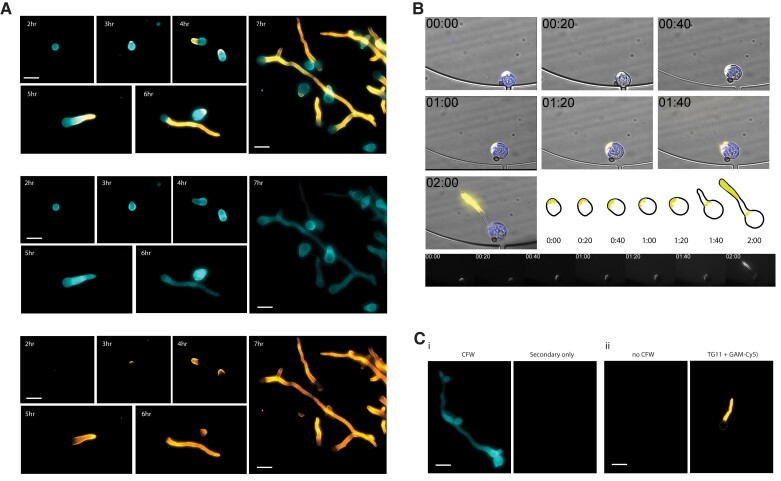
*Rhizopus arrhizus* var *delemar* cells immunostained with mAb TG11 at different stages of spore swelling, germination, and hyphal growth. *A*, Composite photomicrographs (top) and individual channels (middle, bottom) of *R arrhizus* strain 99-880 with DAPI filters for CFW and Cy5 filters for TG11 + GAM-Cy5. Cells are stained with TG11 + GAM-Cy5 (bottom panel) and CFW (middle panel). Representative cells from liquid culture after 2, 3, 4, 5, 6, and 7 hours of incubation were imaged to visualize binding by mAb TG11 at different stages of cell growth. *B*, Time-lapse image series of a single live *R arrhizus* cell grown in media containing CFW and directly conjugated TG11-Cy5 and collected every 20 minutes. Note that indicated times are from the start of imaging with spores pregerminated for 2.5 hours. TG11 is seen to stain the growing hypha, highlighted in the adjacent schematic and Cy5 channel panels below. *C*, Representative negative controls: *i*, CFW, secondary only; *ii*, no CFW, TG11 + GAM-Cy5. Scale bars: 20 μm. CFW, calcofluor white; GAM-Cy5, Cy5-conjugated goat anti-mouse polyclonal IgG; mAb, monoclonal antibody.

To establish the spatiotemporal localization of the TG11 antigen, immunofluorescence microscopy was performed on *R arrhizus* var *delemar* cells at different stages of growth (Figure 1). Indirect and direct staining techniques were used to image fixed and live cells, respectively. The widely used reference strain *R arrhizus* var *delemar* 99-880 was imaged for these experiments, as *R arrhizus* is the most common causative species of mucormycosis. There was no binding of mAb TG11 to ungerminated spores (data not shown). Fixed cells that were collected hourly after spores were allowed to swell for 2 hours showed TG11 staining at the site of polarization (3 hours), on the emerging germ tube (4–6 hours), and localized to hyphae (6–7 hours; Figure 1*A*). Live imaging of spores pregerminated for 2.5 hours revealed that TG11 antigen is first detectable at the tip of the emerging germ tube and then remains detectable along the whole length of the developing hypha (Figure 1*B*, Supplemental Movie 1). Negative controls lacking the primary TG11 mAb showed the absence of nonspecific staining by the secondary antibody (Cy5-conjugated goat anti-mouse polyclonal IgG; Figure 1*Ci*) and no change in TG11 binding in the absence of the chitin-binding dye CFW (Figure 1*Cii*).

### TG11 Antigen Is Detectable on the Hyphal Surface of *Rhizopus*, *Mucor*, *Lichtheimia*, *Cunninghamella*, *Apophysomyces*, *Rhizomucor*, *Saksenaea*, and *Syncephalastrum* Isolates

**Figure 2.**
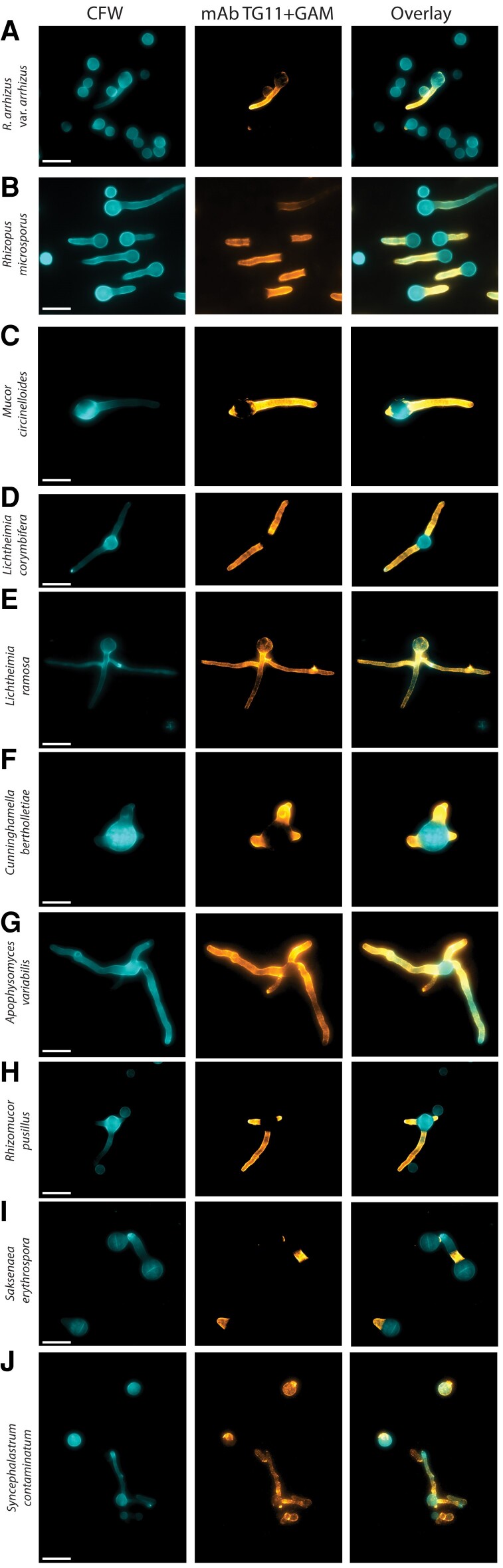
Different mucoralean fungi immunostained with mAb TG11. Composite photomicrographs taken with DAPI and Cy5 fluorescence filters: *A*–*J*, *Rhizopus arrhizus* var *arrhizus* MRL 21E307, *Rhizopus microsporus* FP469-12*, Mucor circinelloides* MRL 22E56177*, Lichtheimia corymbifera* MRL 20E42327*, Lichtheimia ramosa* MRL 21E1059*, Cunninghamella bertholletiae* CBS151.80*, Apophysomyces variabilis* CBS658.93, *Rhizomucor pusillus* MRL 22E19013, *Saksenaea erythrospora* CBS138.279, and *Syncephalastrum contaminatum* MRL 22E22635. Cells are stained with TG11 + GAM-Cy5 (orange) and CFW (blue). CFW, calcofluor white; GAM-Cy5, Cy5-conjugated goat anti-mouse polyclonal IgG; mAb, monoclonal antibody. Scale bar indicates 10 μm.

Indirect immunofluorescence microscopy was performed on an additional 10 mucoralean fungi (Table 1). The TG11 antigen was detected in all 10 isolates (Figure 2).

While TG11 localized to the fungal hyphae, there were some notable differences in antigen distribution in some isolates. *R arrhizus* var *arrhizus*, *Rhizopus microsporus*, *Mucor circinelloides*, *L corymbifera*, *Cunninghamella bertholletiae*, *A variabilis*, and *Rhizomucor pusillus* had a staining pattern similar to that of *R arrhizus* var *delemar*, with homogenous staining along the length of the hyphae and sparingly of the spore body (Figure 2*A*–*D*, 2*F*–*H*). Likewise, staining of *Saksenaea erythrospora* was predominantly located to the hyphae and sparingly of the spore body; however, in cells with longer hyphae, it was apparent that staining did not extend along the whole length of the hyphae but remained localized to the proximal part (Figure 2*I*). *Lichtheimia ramosa* (Figure 2*E*) and *Syncephalastrum contaminatum* (Figure 2*J*) exhibited a more punctate staining pattern, with some variable staining of the spore body. In addition, ungerminated *S contaminatum* spores were observed to exhibit variable morphology, including larger rounded cells that stained with TG11 and CFW, elongated cells that stained with TG11 but not CFW, and small rounded cells that stained with CFW but not TG11 (Figure 2*I*). This suggests species-specific differences in the distribution and exposure of this otherwise hyphal-associated antigen.

### TG11 Antigen Is Localized to the Fungal Cell Wall in Germinating Spores and Hyphae

**Figure 3.**
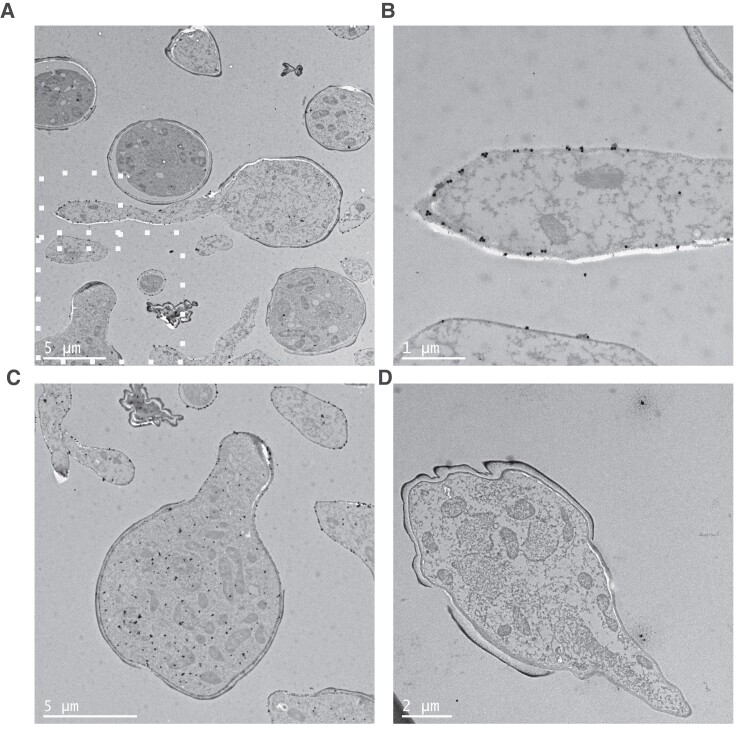
Transmission electron microscopy of immunogold-labeled *Rhizopus arrhizus* var *delemar*. Images of *R arrhizus* var *delemar* stained with (*A–C*) TG11 or (*D*) an IgG2b isotype control and a gold-conjugated secondary antibody. Gold particles are predominantly localized to (*B*) the hyphal cell wall as well as (*C*) the cytoplasm of germinating spores. *B* and *C*, Higher magnifications of the region identified in panel *A* by white squares. Note that panel *C* represents an independent slice of cells imaged in panel *A*.

Together, these observations suggested that TG11 is a cell wall–associated antigen that is enriched in Mucorales hyphae. To gain a more detailed understanding of the location and abundance of the TG11 antigen, the subcellular localization of immunogold-labeled mAb TG11 was visualized in *R arrhizus* var *delemar* by transmission electron microscopy (Figure 3).

Consistent with enrichment of the fluorescent signal along the growing hypha (Figure 1), more gold particles were observed in the hyphal cell wall than in the spore cell wall (Figure 3*A* and 3*B*). The TG11-specific gold density of the hyphal cell wall was 3.96/µm^2^ (isotype control, 0.0548/µm^2^), while the density was 2.71/µm^2^ in the spore body cell wall (isotype control, 0.0606/µm^2^). In addition, an enrichment of gold particles was observed within the cytoplasm of germinating spores (Figure 3*C*), indicative of trafficking of the target antigen within the cell. There was limited nonspecific background and isotype controls (Figure 3*D*). The gold density of the total cell area was 9.94/µm^2^ for the TG11-stained cells as compared with 0.134/µm^2^ for the isotype control and 0.0342/µm^2^ for the background of the TG11-stained section. This was comparable to isotype control background particle density of 0.0420/µm^2^. The hyphal wall and cytoplasmic localization are consistent with previous identification of the TG11 antigen as a secreted polysaccharide [22].

### TG11 Antigen Is Detectable in Hyphae in an Ex Vivo Lung Infection Model

**Figure 4.**
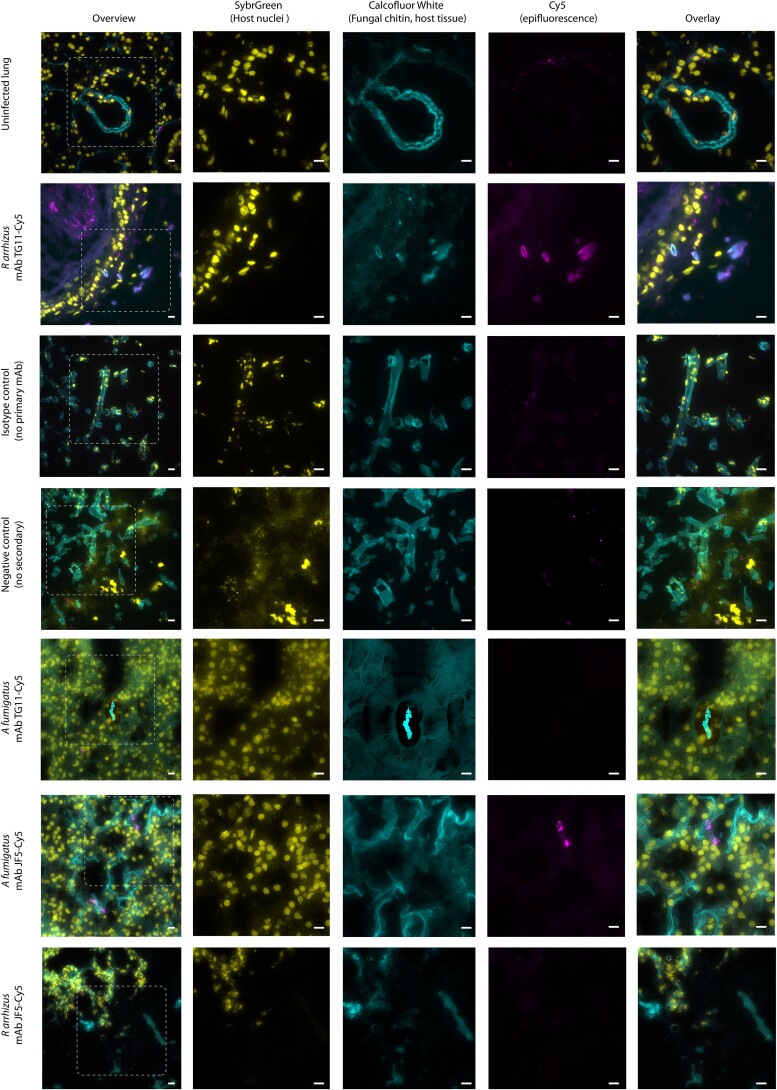
Ex vivo murine lung tissue infected with *Rhizopus arrhizus* var *delemar* and immunostained with mAb TG11. Photomicrographs of murine lung tissue uninfected (first row only) or infected with *R arrhizus* strain 99-880 or *Aspergillus fumigatus* strain A1160p^+^. Images were acquired with FITC (SYBR Green, column 2), DAPI (CFW, column 3), and Cy5 (mAb TG11-GAM; mAb JF5-GAM, column 4) fluorescence filters. Tissue slices are stained as follows: first, second, and fifth rows—mAb TG11 plus GAM-Cy5 (magenta); sixth and seventh rows—mAb JF5 plus GAM-Cy5 (magenta); third row—IgG2b isotype control plus GAM; fourth row—no primary plus GAM alone, CFW for fungal chitin (blue), and SYBR Green for DNA to reveal host cell nuclei (yellow). Note that lung connective tissue and alveolae also stain with CFW but not with mAb TG11-GAM or mAb JF5-GAM. Isotype control is a representative image for both conjugated antibodies. Images acquired in Z-stacks every 0.2 µm are presented as summed Z-projections. Scale bars: 10 μm. CFW, calcofluor white; GAM-Cy5, Cy5-conjugated goat anti-mouse polyclonal IgG; mAb, monoclonal antibody.

To investigate the suitability of mAb TG11 for identification of Mucorales infections in histology sections, murine lungs were infected ex vivo with 10^4^  *R arrhizus* var *delemar* 99-880 spores or *A fumigatus* A1160p^+^ spores. After 24 hours, infected lungs were fixed, sectioned, and stained for epifluorescence microscopy. Fungal hyphae were clearly visible within murine airways by CFW staining. Consistent with in vitro staining, the TG11 antigen was observed along the length of the growing *R arrhizus* hyphae but not *A fumigatus* hyphae, which was instead detected by an *Aspergillus-*specific mAb JF5 (Figure 4) [27].

## DISCUSSION

Here we demonstrate, using immunofluorescence and immunogold electron microscopy, that mAb TG11 detects early- and later-stage hyphal growth of 11 Mucorales fungi. We imaged *R arrhizus* var *delemar* at different stages of development, fixed and over time, and showed that the TG11 antigen is first detectable when polarized to the emerging germ tube and then along the length of the extending hypha. Furthermore, the TG11 antigen is detectable in ex vivo murine lung tissue following invasive infection, and the TG11 mAb is able to differentiate invasive disease caused by Mucorales vs *Aspergillus* species, consistent with previous work demonstrating the specificity of the TG11 mAb for Mucorales fungi [19].

Mucormycosis is caused by a diverse group of >20 fungal species [1–7]. Our study demonstrated that the TG11 antigen is reliably produced by the 11 most common causative species of mucormycosis: *R arrhizus* var *delemar*, *R arrhizus* var *arrhizus*, *R microsporus*, *M circinelloides*, *L corymbifera*, *L ramosa*, *C bertholletiae*, *A variabilis*, *R pusillus*, *S erythrospora*, and *S contaminatum*. The mAb TG11 predominantly bound to hyphae, and immunogold-TG11 transmission electron microscopy of *R arrhizus* var *delemar* revealed abundant antigen in the hyphal cell wall of germinated spores. Together, these results demonstrate the ability of mAb TG11 to detect invasive hyphal growth specifically of mucoralean fungi.

The pathogenesis of mucormycosis is best understood for *R arrhizus*. Infection occurs via inhalation, traumatic inoculation, or contamination of wounds. If not cleared by the immune system, spore germination is followed by hyphal growth, angioinvasion, and tissue necrosis [29, 30]. A biomarker for invasive hyphae may reflect fungal burden and invasive disease. The majority of Mucorales species imaged exhibited similar staining patterns to *R arrhizus* var *delemar*, with sparse staining of spores and homogenous staining along hyphae. Antigen production may therefore correlate well with burden of hyphal growth and invasive disease.

Mucorales are ubiquitous environmental organisms, and their spores can colonize human sinuses, upper respiratory tract, and skin without causing invasive disease [31, 32]. An ideal diagnostic antigen for mucormycosis should differentiate invasive hyphal growth from host colonization or environmental contamination of the clinical sample. The mAb TG11 allows actively germinating spores that produce invasive hyphae to be distinguished from dormant ungerminated spores. While this may not be possible for all fungi, such as *S contaminatum*, where mAb TG11 stained elongated spores, our data demonstrate that it is possible for the most important agents of mucormycosis: *Rhizopus* spp, *Mucor* spp, and *Lichtheimia* spp. Further studies are required to understand the clinical significance of this elongated *S contaminatum* spore morphology, including whether these spores can germinate and cause invasive infection in vivo. In the ex vivo murine lung tissue infection model, TG11 detected invasive *R arrhizus* var *delemar* hyphae. This experiment demonstrates that the fungus produces the TG11 antigen in physiologically relevant growth conditions and in the presence of murine airway immune cells. TG11 may therefore have potential as an immunohistochemistry stain to enhance the detection of mucormycosis in histology samples and to differentiate Mucorales infections from those caused by other fungi, such as *A fumigatus*, the most common mold pathogen of humans.

## CONCLUSIONS

The antigen recognized by mAb TG11 is, to our knowledge, the first potential pan-Mucorales–specific antigen for active Mucorales growth. The next step in evaluating the potential of mAb TG11 for diagnosing mucormycosis should focus on its ability to detect the TG11 antigen in more easy accessible clinical samples, such as serum and urine, to facilitate an earlier diagnosis.

## Supplementary Data

Supplementary materials are available at *The Journal of Infectious Diseases* online (http://jid.oxfordjournals.org/). Supplementary materials consist of data provided by the author that are published to benefit the reader. The posted materials are not copyedited. The contents of all supplementary data are the sole responsibility of the authors. Questions or messages regarding errors should be addressed to the author.

## Supplementary Material

jiae375_Supplementary_Data

## Notes

***Acknowledgments***. We thank Dr Christian Hacker and Dr Lenka Koptasikova at the Exeter University Bioimaging Centre and Gillian Milne and the Microscopy and Histology Facility, University of Aberdeen, for help with transmission electron microscopy sample preparation and imaging. We acknowledge the patients and Professors Andrew Borman and Elizabeth Johnson at the UK Health Security Agency National Collection of Pathogenic Fungi, Bristol, UK, for providing clinical isolates. We gratefully acknowledge our funders.

***Author contributions***. A. C. H., D. E. C.-L., A. W., E. R. B.: conceptualization; A. C. H., D. E. C.-L., I. K., E. R. B.: investigation, validation, data curation, visualization, formal analysis. A. C. H., D. E. C.-L., E. R. B.: writing–original draft. A. C. H., D. E. C.-L., I. K., D. W., C. R. T., A. W., E. R. B.: writing–review and editing. D. W., C. R. T., A. W., E. R. B.: resources, supervision, funding acquisition.

***Disclaimer.*** Although this study was supported by the NIHR Exeter Biomedical Research Centre, additional work may have been undertaken by the University of Exeter Biological Services Unit. The views expressed are those of the authors and not necessarily those of the National Institute for Health and Care Research or the Department of Health and Social Care. The funders had no role in study design, data collection and interpretation, or the decision to submit the work for publication.

***Financial support***. We are grateful for support from Noah’s Pink Balloon Leukaemia Fund (fellowship to A.C.H.). This work was supported by the Medical Research Centre for Medical Mycology ((MR/N006364/2) and the NIHR Exeter Biomedical Research Centre (MR/N006364/NIHR); the UK Biotechnology and Biological Sciences Research Council and the USA National Science Foundation (BB/W002760/1 to D. E. C.-L. and E. R. B.); a Wellcome Trust Senior Research Fellowship (214317/A/18/Z to I. K. and D. W.); a Sir Henry Dale Fellowship jointly funded by the Wellcome Trust and the Royal Society (211241/Z/18/Z to E. R. B.); and the Lister Institute (to E. R. B.).
